# A weak allele of *OsNRAMP5* for safer rice

**DOI:** 10.1093/jxb/erac323

**Published:** 2022-10-18

**Authors:** Fang-Jie Zhao, Jia-Dong Chang

**Affiliations:** State Key Laboratory of Crop Genetics and Germplasm Enhancement, College of Resources and Environmental Sciences, Nanjing Agricultural University, Nanjing 210095, China; State Key Laboratory of Crop Genetics and Germplasm Enhancement, College of Resources and Environmental Sciences, Nanjing Agricultural University, Nanjing 210095, China

**Keywords:** Cadmium, food safety, manganese, OsNRAMP5, rice

This article comments on:

**Kuramata M, Abe T, Tanikawa H, Sugimoto K, Ishikawa S**. 2022. A weak allele of *OsNRAMP5* confers moderate cadmium uptake while avoiding manganese deficiency in rice. Journal of Experimental Botany **73**, 6475–6489.

**Rice is the staple food for over half of the world’s population. Unfortunately, its propensity to accumulate the toxic metal cadmium (Cd) also makes it a major dietary source of Cd (** Zhao *et al.*, 2022 **). Reducing Cd accumulation in rice is an important task for improving food safety, as soil Cd contamination is an increasing problem worldwide. Using the TILLING (targeting induced local lesions in genomes) method,** Kuramata *et al.* (2022 **) identified a mutant allele, *OsNRAMP5-*Q337K, with reduced accumulation of both Cd and manganese (Mn), the latter being the physiological substrate of the OsNRAMP5 transporter protein (** Sasaki *et al.*, 2012 **). This weak allele may be useful for breeding rice cultivars with reduced Cd accumulation without causing severe Mn deficiency.**

## The dilemma between Mn and Cd

NRAMPs (natural resistance-associated macrophage proteins) are a family of membrane transporters for divalent transition metals. NRAMP transporters are present in all organisms, including bacteria, fungi, plants, and animals (Bozzi and Gaudet, 2021). The first *NRAMP* gene (*NRAMP1*) was cloned from mice varying in natural resistance to infection with intracellular pathogens (Vidal *et al.*, 1993). *NRAMP1* encodes a divalent metal efflux pump at the phagosomal membrane of macrophages that functions to extract essential metals such as Mn^2+^ and Fe^2+^ from phagosomes to help kill engulfed pathogens (Forbes and Gros, 2001; Bozzi and Gaudet, 2021). *NRAMP2* (also called *DCT1* or *DMT1*) was cloned from rat and encodes a transporter for the uptake of Fe^2+^ and other divalent transition metals in the proximal duodenum of the small intestines (Gunshin *et al.*, 1997). The genome of rice contains seven *NRAMP* genes, some of which have been characterized functionally (Table 1). Most mammalian and prokaryotic NRAMP transporters are promiscuous regarding their transport substrates, often including Mn^2+^, Fe^2+^, Co^2+^, Ni^2+^, Zn^2+^, and Cd^2+^ (Gunshin *et al.*, 1997; Bozzi and Gaudet, 2021) Mn^2+^, Fe^2+^, and Cd^2+^ are also the common substrates for plant NRAMPs (Table 1), except OsNRAMP4 (OsNRAT1) which unusually transports Al^3+^ (Xia *et al.*, 2010). While Mn, Fe, Zn, Co, and Ni are essential for life, Cd is highly toxic and a carcinogen to humans. The reason why evolution has not resulted in Cd-discriminating NRAMPs may be because Cd usually is not present at levels in the environment high enough to exert a strong and persistent selective pressure on organisms to evolve transporter proteins that can discriminate it from other essential trace metals (Bozzi and Gaudet, 2021; Zhao *et al.*, 2022).

**Table 1.** Functions of NRAMPs in rice (*Oryza sativa*).

NRAMP family	Location	Transport substrates	Functions	References
OsNRAMP1	Plasma membrane	Mn, Cd	Uptake of Mn and Cd	Takahashi *et al.* (2011); Chang *et al.* (2020)
OsNRAMP2	Tonoplast	Fe, Cd	Exporting Fe and Cd from the vacuole to the cytosol	Li *et al.* (2021); Chang *et al.* (2022)
OsNRAMP3	Plasma membrane	Mn	Distribution of Mn in the nodes	Yamaji *et al.* (2013)
OsNRAMP4(OsNRAT1)	Plasma membrane	Al	Uptake of Al	Xia *et al.* (2010)
OsNRAMP5	Plasma membrane	Mn, Cd	Uptake of Mn and Cd	Ishikawa *et al.* (2012); Sasaki *et al.* (2012)
OsNRAMP6	Plasma membrane	Fe, Mn	Negatively regulates resistance to the rice blast fungus in rice plants	Peris-Peris *et al.* (2017)

Ten years ago, OsNRAMP5 was identified as the major transporter for the uptake of Mn and Cd in rice (Ishikawa *et al.*, 2012; Sasaki *et al.*, 2012). OsNRAMP5 is polarly localized to the distal side of the plasma membranes of the exodermal and endodermal cells of rice roots responsible for transporting Mn^2+^ and Cd^2+^ into the root cells (Sasaki *et al.*, 2012). Knockout of *OsNRAMP5* resulted in dramatic decreases, often >90%, in the plant uptake of Mn and Cd and their accumulation in the grains (Ishikawa *et al.*, 2012; Sasaki *et al.*, 2012; Yang *et al.*, 2014). Because Mn is an essential micronutrient functioning, among others, in PSII, knockout mutants of *OsNRAMP5* grew poorly under conditions of low Mn supply and exhibited typical Mn deficiency symptoms (Sasaki *et al.*, 2012; Yang *et al.*, 2014; Chang *et al.*, 2020). The growth defect can be rescued by supplying a relatively high concentration of Mn in the nutrient solution (8 μM) (Yang *et al.*, 2014). When paddy soils are flooded for growing rice, the anaerobic conditions in the soils favour the reduction of manganese oxides, resulting in the mobilization of Mn into the soil solutions. The concentration of soluble Mn in soil solutions is highly variable, ranging from sub-micromolar to several millimolar, depending on the soil type and the redox potential (Wang *et al.*, 2019). Where soil Mn availability is sufficiently high, knockout mutants of *OsNRAMP5* can grow normally without yield penalty and offer the great advantage of a dramatically reduced Cd accumulation in the grain (Ishikawa *et al.*, 2012). An irradiation-induced rice mutant with a mutated allele of *OsNRAMP5* has been registered as a new low-Cd cultivar in Japan (Ishikawa, 2020). The CRISPR/Cas9 gene editing technique has also been used to knock out *OsNRAMP5* to generate low Cd-accumulating rice (Tang *et al.*, 2017).

However, given that Mn availability varies widely among soils, a total knockout of *OsNRAMP5* represents a risky strategy for combating the Cd contamination problem. Under field conditions, the low-Cd *OsNRAMP5* knockout mutant was found to be more susceptible to brown spot disease than the parental variety (Ishikawa, 2020). Moreover, *OsNRAMP5* knockout mutants were less tolerant of high temperatures at the flowering stage, resulting in 20–30% yield losses (Dong *et al.*, 2021). These results suggest that, apart from affecting plant growth, Mn may also play an important role in plants’ resistance to biotic and abiotic stresses.

## A weak transporter offers a compromise solution

To circumvent the above problems, Kuramata *et al.* (2022) looked for mutant alleles of *OsNRAMP5* that may lead to lower Cd uptake but without causing severe Mn deficiency. They identified several mutant alleles from a mutagenized pool of rice. Among these alleles, one with a substitution of glutamine at the 337th position by lysine (Q337K) in the protein sequence showed a Cd and Mn accumulation phenotype intermediate between that of the wild type and the knockout lines. Heterologous expression in yeast and physiological studies of the Q337K mutant confirmed that this mutation significantly weakened, but did not abolish, the transport activities of OsNRAMP5 for both Mn^2+^ and Cd^2+^. The *OsNRAMP5-*Q337K mutant was less susceptible to Mn deficiency than the knockout lines and could tolerate an Mn concentration in the nutrient solution as low as 0.1 μM without showing Mn deficiency symptoms in a hydroponic experiment. When grown in a paddy field with a Cd-contaminated soil, the *OsNRAMP5-*Q337K mutant produced 50% and 30% lower grain concentrations of Cd and Mn, respectively, than the wild type, whereas grain yields were comparable. Thus, the *OsNRAMP5-*Q337K mutant is a compromise between acquiring enough Mn and not accumulating too much Cd.

## How to increase substrate selectivity?

**Fig. 1.**
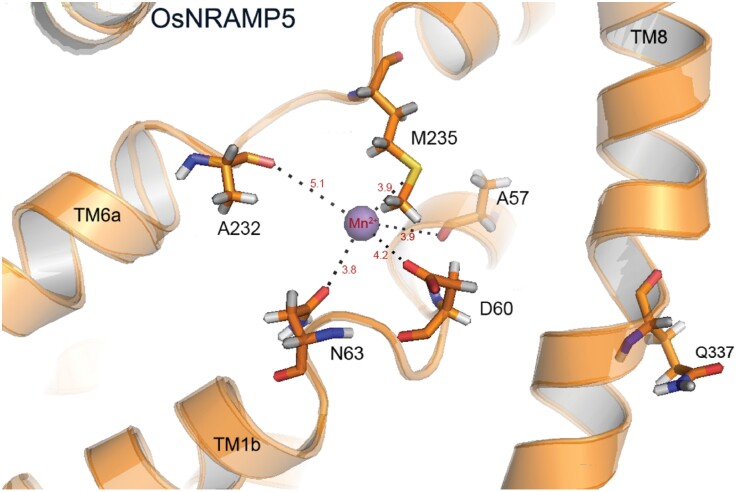
The metal-binding site of OsNRAMP5 based on homology modelling using the protein structure of *Staphylococcus capitis* NRAMP (PDB ID: 5M95). The amino acid residues A57, D60, and N63 in TM1, and A232 and M235 in TM6, provide coordination to the metal ion. Q337 in TM8, reported by Kuramata *et al.* (2022) as important for the transport rate, may affect the conformational dynamics of the protein.

An ideal solution to solve the problem of Cd contamination in rice would be to enhance the selectivity of OsNRAMP5 towards Mn^2+^. While the *OsNRAMP5-*Q337K allele decreases the uptake of both Mn^2+^ and Cd^2+^, it does not affect the transporter’s selectivity towards these two competing substrates (Kuramata *et al.*, 2022). In fact, substitutions of Q337 with other amino acids affect the transport activities of Mn^2+^ and Cd^2+^ similarly, suggesting that the 337th residue affects the rate of transport but not the substrate selectivity (Kuramata *et al.*, 2022). Q337 is located in transmembrane helix 8 (TM8), which is not directly involved in metal binding (Bozzi and Gaudet, 2021; Fig. 1). Based on homology modelling of the protein structure, Kuramata *et al.* (2022) suggest that amino acid substitutions of Q337 affect the conformational dynamics of the protein by changing the structural flexibility of TM8 and the stability of the loop structure between TM7 and TM8.

Studies on the structures of mammalian and prokaryotic NRAMPs have yielded a wealth of information regarding the transport mechanisms of these proteins (Ehrnstorfer *et al.*, 2014; Bozzi and Gaudet, 2021). The highly conserved metal-binding site is located within TMs 1 and 6. Based on homology modelling, five amino acid residues in OsNRAMP5, namely A57, D60, and N63 in TM1, and A232 and M235 in TM6, provide coordination to the metal ion (Fig. 1). Interestingly, the methionine residue in TM6 plays a crucial role in metal transport and substrate selectivity. In the NRAMP of the bacterium *Deinococcus radiodurans*, the presence of methionine (M230, corresponding to M235 in OsNRAMP5) makes Cd^2+^ the preferred substrate. When M230 was mutated to alanine (A), Cd^2+^ uptake decreased greatly but uptake of Mn^2+^ and Fe^2+^ was still robust, although this mutation also allowed Ca^2+^ and Mg^2+^ to be transported (Bozzi *et al.*, 2016). The importance of methionine for Cd^2+^ transport is consistent with the ‘soft’ metal Cd preferring S-containing ligands. M235 in OsNRAMP5 may be a target to manipulate the selectivity between Mn^2+^ and Cd^2+^. A potential pitfall is that Ca^2+^ and Mg^2+^, which are usually abundant in soils, may then compete with Mn^2+^ uptake (Bozzi *et al.*, 2016).

Apart from the metal-binding site, mutations in other amino acid residues may also affect substrate selectivity. An elegant study was presented by Pottier *et al.* (2015), who randomly mutated cDNA of the Arabidopsis *AtNRAMP4*, which encodes a tonoplast efflux transporter with transport activities for Fe^2+^, Mn^2+^, Zn^2+^, and Cd^2+^. They then screened the mutated *AtNRAMP4* in the yeast mutant *fet3fet4* defective in Fe uptake for mutant alleles that could rescue the growth of the mutant (i.e. restoring Fe^2+^ uptake) but with a suppressed sensitivity to Cd (i.e. decreased Cd^2+^ uptake). They found that the mutations L67I, L67V, E401K, and F413I restored Fe^2+^ uptake but with suppressed Cd sensitivity compared with the wild-type *AtNRAMP4*. Further experiments showed that L67V and L67I mutations specifically impaired Cd^2+^ transport by AtNRAMP4. These results provide a proof of concept that NRAMPs could be manipulated to discriminate Cd^2+^ while maintaining the transport activities for essential trace metals. It would be interesting to evaluate whether mutations in the corresponding positions in OsNRAMP5 produce the same effects.

While the study of Kuramata *et al.* (2022) represents a step forward in the direction towards low Cd rice, a more perfect solution by altering metal substrate preference awaits further investigations.
